# Safety of DW-MSC infusion in patients with low clinical risk COVID-19 infection: a randomized, double-blind, placebo-controlled trial

**DOI:** 10.1186/s13287-022-02812-4

**Published:** 2022-04-01

**Authors:** Muhammad Karyana, Irawaty Djaharuddin, Lutfah Rif’ati, Mansyur Arif, Mi Kyung Choi, Nova Angginy, Aeri Yoon, Jumi Han, Fonny Josh, Dona Arlinda, Asvin Narulita, Faisal Muchtar, Rizki Auliah Bakri, s Irmansyah

**Affiliations:** 1 National Institute of Health and Research Development, Ministry of Health, Republic of Indonesia (NIHRD, MoH RI), Jakarta, Indonesia; 2 RSUP Dr. Wahidin Sudirohusodo, Pulmonology and Respiratory Medicine, Medical Faculty, Hasanuddin University, Makassar, Indonesia; 3 RSUP Dr. Wahidin Sudirohusodo, Makassar, Indonesia; 4 Daewoong Pharmaceutical Co Ltd., Seoul, Republic of Korea; 5 Daewoong Infion, Jakarta, Indonesia

**Keywords:** COVID-19, SARS-CoV-2, Mesenchymal stem cells, Cytokines, Adverse events, Safety

## Abstract

**Background:**

Due to their immunomodulatory properties, mesenchymal stem cells (MSCs) have been proposed to have therapeutic potential to improve clinical outcomes in COVID-19. However, the safety and efficacy profile of MSC infusion therapy in patients with non-severe COVID-19 infection has not been completely established; there is, in particular, a substantial void in the literature on dose-dependent studies of MSC infusion in patients with low clinical risk COVID-19 infection.

**Methods:**

This phase 1 double-blind, placebo-controlled, randomized clinical trial examines the safety, feasibility, and tolerability of 2 doses (high and low) of DW-MSC in patients with low clinical risk COVID-19. A total of 9 patients were enrolled in this study and randomized into low-dose (TL), high-dose (TH), and placebo (C) groups. Subjects in the TL and TH groups received single intravenous infusions of 5.0 × 10^7^ cells and 1.0 × 10^8^ cells, respectively. The main outcome was the occurrence of treatment-emergent adverse events (TEAE) during the 28-day study period. Vital signs and various inflammatory markers were also monitored weekly during the observation period.

**Results:**

There were no apparent differences in clinical characteristics between study groups (TL, TH, and C) at baseline. All patients did not show the progression of severity during the study period. During the course of the study, 6 episodes of TEAE were observed in 5 subjects; however, none of the TEAEs were severe. During the follow-up period, 8 subjects recovered and were discharged from the hospital without complications. A subject exhibited abnormal liver function biomarkers at the end of the study period. Changes in inflammatory markers throughout the clinical course were not vastly different across study groups.

**Conclusions:**

Our clinical trial has provided reliable results regarding the safety of MSCs in low clinical risk COVID-19 subjects treated with MSCs. However, further confirmation of the therapeutic efficacy aspects of MSC will require large-scale randomized controlled trials in subjects with varying severity profiles for COVID-19.

***Trial registration*:**

ClinicalTrials.gov, NCT04535856. Registered 2 September 2020, https://clinicaltrials.gov/ct2/show/NCT04535856

**Supplementary Information:**

The online version contains supplementary material available at 10.1186/s13287-022-02812-4.

## Introduction

Stem cell therapies are emerging as promising treatment modalities that can reduce inflammation and restore lung damage caused by corona virus disease of 2019 (COVID-19), alone or in combination with existing therapy regimens [1, 2]. An increasing body of research indicates that mesenchymal stem cell (MSC)-based treatment can help manage acute respiratory distress syndrome (ARDS) due to the ability of MSCs to produce anti-inflammatory, antifibrosis, and anti-apoptosis cytokines [3–5], promote recovery of lung function, and potentially influence the progression of pulmonary fibrosis [6, 7]. As a result, MSC therapies may help in treating pneumonia, inflammation, and sepsis, which are among the leading causes of morbidity and death in patients with COVID-19.

For different disorders such as lung diseases [2, 8], bronchopulmonary dysplasia [9], cardiovascular diseases [10], diabetes mellitus [11, 12], and spinal cord injury [13], the safety profile and efficacy of MSCs are relatively well established. Although in the context of COVID-19, the safety and efficacy of MSCs have not been completely proven, some pilot and phase 1 studies [5, 14, 15] reported no allergic responses related to immediate infusion, delayed hypersensitivity, or subsequent infections. Improvements in clinical outcomes, including oxygenation index (OI) and inflammation markers, have also been reported [5, 14]. Similar results have been reported from other phase 1 and 2 double-blind, randomized controlled trials, showing no serious adverse events occurred during the studies, and in general, the infusion of MSCs has been reported to improve patient survival and reduce inflammation [16, 17].

Due to the differences in the research designs used in these prior clinical studies, it is difficult to generalize the reported findings. In particular, the number of doses and the number of cells in a single administration have been extensively varied from 0.5 × 10^6^ cells/kg body weight to 400 × 10^6^ cells/dose [18]. There is little understanding of the optimal or safe doses of MSCs applied in patients with different severity of COVID-19. In particular, most previous studies were conducted in severely ill COVID-19 patients and were uncontrolled or non-randomized, reflecting the need for a more controlled study. It would be interesting to see whether the use of MSC treatment in patients with low clinical risk COVID-19 infection can help them recover faster or if it can help them avoid progressing to a more severe infection that is difficult to manage. However, to date, no studies have been conducted to determine the impact of different doses of MSC on safety and tolerance in the context of COVID-19, nor is there much evidence available on the practicality, safety, and effectiveness of MSC infusion in patients with low clinical risk COVID-19.

The current study presents the results of the phase 1 double-blind, placebo-controlled randomized clinical trial that compares the safety profiles of single low- and high-dose MSC infusions in patients with low clinical risk COVID-19.

## Methods

### Trial design and ethical consideration

This was a phase 1 randomized, double-blind, placebo-controlled clinical trial to evaluate the safety and investigate the efficacy measures of a single dose of intravenous DW-MSCs in patients with COVID-19 (clinicaltrials.gov identifier NCT04535856). Ethical clearance (LB.02.01/KE.443/2020) was obtained from the National Institute of Health and Research Development Ethics Committee (NIHRD EC). All patients signed their written consent before the screening procedure. Patients could voluntarily withdraw from the trial, and the investigator can discontinue the participation of the patients at any time.

### DW-MSCs

DW-MSCs were manufactured by the National Institute of Health, National Center for Stem Cell and Regenerative Medicine (Osong, Korea) from embryonic stem cells (SNUhES35) in a Good Manufacturing Process (GMP) grade facility. A master working cell bank (MWCB) vial (passage 8) was thawed and inoculated in a T175 cm^2^ flask with MSC culture medium (serum-free, xeno-free). Cells were expanded through 3 times of passages using T175 cm^2^ and HYPERFlask. At passage 12, these cultured cells were harvested, frozen, and stored in LN_2_. DW-MSCs is characterized by the expression of cell surface markers such as CD29, CD44, CD73, CD105, but it does not have hematopoietic lineage markers of CD34 and CD45, no HLA-DR immune response marker according to the International Society for Cell & Gene Therapy (ISCT) standard, and no SSEA-3, TRA-1–60, and TRA-1–81 cell markers. DW-MSCs can differentiate into trilineage cells (chondroblasts, osteoblasts, and adipocytes). The quality of the DW-MSCs has been confirmed through release tests, including sterility tests, endotoxin, mycoplasma, and adventitious virus tests.

### Preclinical evaluation of the safety and efficacy of DW-MSC

There were no signs of tumorigenesis in 6 months following a single intravenous (2.5 × 10^8^ cells/kg) administration of DW-MSCs to nude mice. In addition, no adverse/toxic reactions such as changes in the general symptoms, body weight, autopsy, and histopathological examination, nor death were observed in 13 weeks after intravenous administration with various concentrations (5.0 × 10^7^ cells/kg, 1.25 × 10^8^ cells/kg, 2.5 × 10^8^ cells/kg) of DW-MSC. However, immediately after administration of doses of DW-MSC of 1.25 × 10^8^ cells/kg and 2.5 × 10^8^ cells/kg in females and 2.5 × 10^8^ cells/kg in males, convulsions, dyspnea, loss of spontaneous movement, and gait abnormalities persisted for several days with thrombosis in the atrial and ventricular of the heart and pulmonary arteries on biopsy were evident. In conclusion, no adverse effect level (NOAEL) of DW-MSC was determined to be 1.25 × 10^8^ cells/kg for males and 5.0 × 10^7^ cells/kg for females. The dose of DW-MSC for COVID-19 patients was established based on the lowest concentration, of which safety was confirmed in animal models among doses below NOAEL. The lowest efficacious dose was considered the lowest concentration of DW-MSCs at which the virus titer in the nasal fluid of the COVID-19 ferret model decreased significantly compared to the control group. It was found to be 1.67 × 10^6^ cells/kg; notably, this dose is 3.3% of the NOAEL in the female nude mouse group or 1.3% in the male nude mouse group. As the average body weight in humans was considered 60 kgs, the high dose for this study was established at 1 × 10^8^ cells (1.67 × 10^6^ cells/kg × 60 kgs), and the low dose was established at half the high dose (5 × 10^7^ cells).

### Selection of trial subjects

**Fig. 1:**
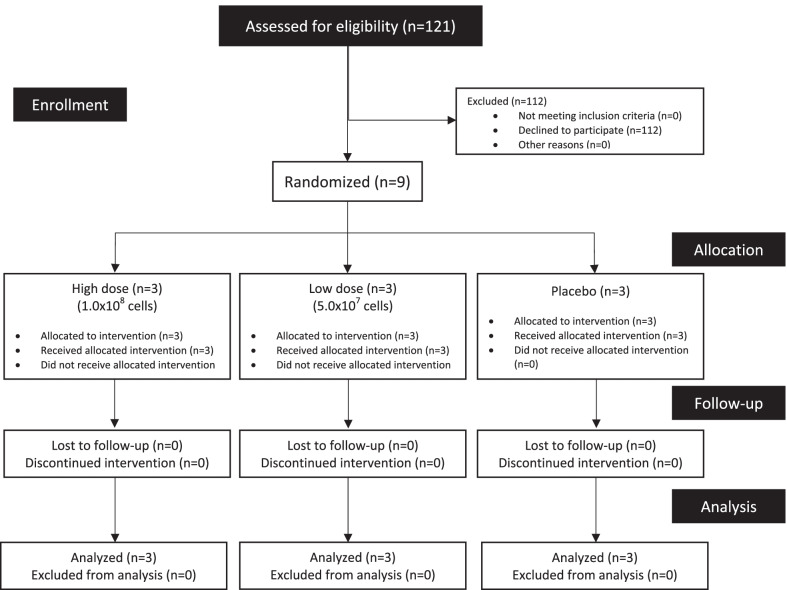
Enrolment, randomization, and study flow diagram

The study was carried out at Dr. Wahidin Sudirohusodo Hospital, Makassar, from August 2020 to March 2021. Inclusion criteria were COVID-19 confirmed by real-time polymerase chain reaction (RT-PCR), 19 years or older, and clinically mild according to the National Early Warning Score 2 (NEWS2). Patients, who had a history of hypersensitivity to the components of the investigational products (IP), had viral or bacterial pneumonia other than expected indication, received organ transplants within 6 months of screening, had a history of pulmonary embolism, had underlying diseases that may obtain the benefit from the IP, participated in other clinical studies, and had life expectancy less than 24 h, were excluded. Pregnant and lactating patients were also excluded. The time of physical examination, clinical laboratory tests, and other medical examinations are listed in the protocol flowchart (Fig. 1, Additional file 1: Table S1).

### Concealment and randomization

The trial was carried out under double-blind conditions. After signing the consent form, patients who met the final inclusion and exclusion criteria were randomized to the test groups (low dose (TL) and high dose (TH)) or control (C)) in a ratio of 1:1:1.

### Administration of DW-MSCs

The sponsor manufactured the IP and then packaged and supplied to the clinical trial pharmacists at the study institution. The IP was stored separately in a safe and restricted place, at − 80 °C. During administration, DW-MSCs were mixed in a sap bag containing 100 ml of 10% dextrose (glucose) depending on the assigned dose; and then massaged appropriately to prepare the cells to be evenly suspended. The prepared suspension is injected into the subjects by intravenous infusion. The cell viability was ascertained to be more than 90%. The infusion time was approximately 30–45 min at a speed of approximately 50 drops/min. The vehicle solution was also tested for 14-day sterility, Gram stain, and endotoxin.

### Interventions

Patients were assigned to receive infusions: TL (5.0 × 10^7^ cells), TH (1.0 × 10^8^ cells), or C (Cryostor CS10 containing dimethyl sulfoxide 10%, sucrose 1%, sodium hydroxide 0.6%, and potassium hydroxide 0.168%). All subjects received standard medications according to their conditions following the current institutional guidelines of COVID-19. DW-MSCs was an adjunct to standard therapy.

Laboratory test items included hematology parameters, serum chemistry, serum coagulation, urinalysis, and cytokines (IL-6, TNF-α, IL-1β, IFN-γ) (MilliporeSigma, Merck KGaA, Darmstadt, Germany); the details are listed in Table 2. A protein array SIGMA ELISA Kit (Sigma-Aldrich, MO, USA) was used to determine plasma levels of interferon IFN-γ, IL-1β, IL-6, and TNF-α. The analysis was carried out according to the manufacturer's instructions.

All laboratory test results, including screening tests, use only the test results performed at the visit. Inflammatory markers were tested before (random) assignment and subsequently on visit days (7, 14, and 28).

### Outcomes

#### Primary endpoints

The primary endpoint was the incidence of treatment-emergent adverse events (TEAE) during the observation period. TEAE was defined as undesirable events not present prior to study treatment or an already present event that worsens either in intensity or frequency following the study treatment. The TEAE report was organized according to the system organ class and the preferred term using the Medical Dictionary for Regulatory Activities (MedDRA). The investigators assessed the severity of each adverse event according to the National Cancer Institute Common Terminology Criteria for Adverse Events (NCI-CTCAE), version 5.0. In the event of a severe reaction or embolization, the study would be terminated.

#### Secondary endpoints

Secondary endpoints were survival rate at each study visit, duration of hospitalization, and clinical improvement. For the duration of hospitalization, viral shedding as measured by the RT-PCR results was used as the parameter to discharge subjects from the hospital to the quarantine house, where subjects stayed until the end of the study.

### Statistical analysis

Descriptive statistics (minimum and maximum) were presented for continuous variables, when appropriate.

## Results

### Patients

Of the 121 COVID-19 patients screened for this study, 112 patients were unwilling to be monitored in the hospital for 28 days. From November 10 to December 17, 2020, 9 subjects were enrolled and randomly assigned to 1 of the 3 groups: TL, TH, and C. All patients received the same standard therapies, which were oseltamivir and azithromycin.

The baseline characteristics of each subject, including age, sex, NEWS2, coexisting disease, vital sign abnormality, and chest X-ray abnormality, are presented in Table 1. All subjects were distributed between the ages of 31 and 47, and a total of 9 subjects were included, 1 female and 8 males. According to the aggregate score, NEWS2 was similar (0–1) in the 3 dosing groups, and all subjects were at low risk. Most of the subjects were prehypertensive. A chest X-ray abnormality was found in 2, 1, and 1 of the subjects in the TH, TL, and C groups, respectively. Lobar pneumonia was detected in 1 subject who received a high dose and another who received a low dose of DW-MSCs. All subjects were hospitalized, but none needed oxygen supplementation (score 3, WHO ordinal scale for clinical improvement). All subjects had respiratory rates of less than 20 breaths per minute, their SpO_2_ levels were equal/ greater than 96%, and PaO_2_/FiO_2_ ratio was greater than 399 mmHg. Laboratory data were within normal limits from the time of admission until the time of the cell infusions.

**Table 1:** Baseline characteristics of enrolled patients and treatment-emergent adverse event

	TH, *n* = 3			TL, * n* = 3			Placebo, * n* = 3		
	TH1	TH2	TH3	TL1	TL2	TL3	C1	C2	C3
Age (years)	47	32	31	32	38	33	39	34	43
Sex	Male	Male	Male	Male	Male	Male	Female	Male	Male
National Early Warning Score 2 (NEWS2)	0	0	0	0	0	1	0	0	1
Co-existing disease						Conjunctivitis	Thyroid enlargement		
Vital signs abnormality	Pre-Hyper-tension	No	Pre-Hyper-tension	Pre-Hyper-tension	Pre-Hyper-tension	Pre-Hyper-tension	Pre-Hyper-tension	Pre-Hyper-tension	Stage 1 Hyper-tension
Chest X-ray abnormality	No	Bilateral hilar lymphadenopathy	Left lobe pneumonia	Right lobe pneumonia	No	No	No	No	Aorta dilatation
Electrocardiography (ECG) abnormality	No	No	No	No	No	No	No	No	No
Laboratory abnormality								High level of AST/ALT	
Treatment emergent adverse event (TEAE)	Right lobe pneumonia	–	Rash maculo-papular	Blood lactate	–	–	Cutaneous candidiasis, myalgia	Hepato-biliary disorder	–
Starting day (duration in days) of TEAE	7*	–	9 (6)	3 (Unknown)	–	–	14 (10; 2)	0 (> 44)	–

*TH* treatment high-dose group, *TL* treatment low-dose group, *C* placebo group

*The first day TEAE was detected following first scheduled chest X-ray (at days 7); the duration was unknown, but no sign of pneumonia was detected following second scheduled chest X-ray examination (at days 14)

### Clinical course after cell infusion

#### Primary endpoint

TEAE did not occur during MSC infusion and within 24 h after infusion. We noted that all 9 treated subjects tolerated DW-MSC infusions, and there were no severe adverse events related to infusions.

Table 1 describes the 6 TEAE in 5 subjects after infusion. TH1 showed pneumonia in the right lobe based on his chest X-ray on the routine procedure on day 7 but was normal on the following examination on day 14. TH3 showed a maculopapular rash on day 9 that lasted 6 days. TL1 had an increase in blood lactate level (6.5 mmol/dL), observed on day 3 during routine standard of care laboratory tests; however, no follow-up tests were performed. C1 experienced cutaneous candidiasis and myalgia on day 14 that lasted 10 and 2 days, respectively, while C2 showed worsening of abnormal liver function biomarkers 7–14 days after enrollment, which did not resolve till day 28 (Table 1).

The 9 subjects recovered and were discharged from the hospital during the follow-up period without a fatality. Severe acute respiratory syndrome coronavirus 2 (SARS-CoV-2) was not detected on days 5, 8, and 16 in subjects in the TH group, days 6, 13, and 26 in subjects in the TL group, and days 5, 5, and 14 in the C group. As negative SARS-CoV-2 RT-PCR results were used as criteria for discharge from the hospital, these were also the days when the WHO ordinal scale for clinical improvement changed from 3 (hospitalized, no oxygen therapy needed) to 1 (no limitation of activities). On day 14, 7 of 9 subjects had an ordinal scale improved from 3 to 1, and on day 28, all subjects had the ordinal scale of 1. None of the subjects had limitations in physical activity (ordinal scale of 2) during the study period.

NEWS2 at baseline and on day 28 was 0 in all subjects. On day 7, NEWS2 was 1 in 4 subjects, as these subjects had body temperature between 35.1–36.0 °C. NEWS2 in subject C3 deteriorated to 2 on day 14 as oxygen saturation dropped to 94–95% and heart rate was between 91 and 110/min, but NEWS2 was back to 0 on day 28. In the other subjects, the NEWS2 on days 7, 14, and 28 was 0.

The PaO_2_/FiO_2_ ratio was within the normal value in all subjects at the beginning of the study. None of the subjects had a PaO_2_/FiO_2_ ratio < 400 mmHg on days 3 and 28 of observation. Only 1 subject had a PaO_2_/FiO_2_ ratio < 300 mmHg on day 7 (TL1) and on day 10 (TH1), which increased to normal levels at subsequent follow-up (Additional file 1: Fig. S1). A subject in the TL group had a PaO_2_/FiO_2_ ratio of 381 mmHg on day 14 but returned to normal on day 28 (486 mmHg).

Two subjects (TH3 and TL1) had lobar pneumonia on 1 side at the beginning of the study that improved on subsequent visits. One subject (TH1) had pneumonia in the right lobe on day 7 that was not seen on chest X-ray subsequent visits. No pneumonia was detected in the chest X-ray of any subject on days 14 and 28 of the visit.

Table 2 and Additional file 1: Figure S2 shows the changes in the leukocyte count, lymphocyte fraction, ESR, CRP, fibrinogen, IL-6, TNF-α, IL-1β, and IFN-γ on days 1, 7, 14, and 28 following infusion. All subjects had normal leukocyte counts at baseline and at all study visits, while increased lymphocyte (%) was found in TH3 on day 28, TL3 on day 7 and day 28, C2 on day 14, and C3 on day 28. The ESR slightly elevated in TL1 on day 28 (30 mm/h), which was consistent with his high level of CRP (38.5 mg/L) and fibrinogen (476.1 mg/dL) level. Similarly, TH2 and TL3 showed elevated CRP at baseline, and C3 showed elevated fibrinogen at baseline.

**Table 2:** Laboratory parameters in subjects with/without MSCs infusion on day 1, 7, 14, and 28

	High dose, * n* = 3			Low dose, * n* = 3			Placebo, * n* = 3		
	TH1	TH2	TH3	TL1	TL2	TL3	C1	C2	C3
*White blood cell, /μL *(*normal range*: 4–10 × 10^3^/μL)									
Day 1	5300	5900	7800	8200	7600	7600	4900	7500	5300
Day 7	4100	7200	7700	7400	8800	5100	4800	7300	7100
Day 14	5700	7700	5900	6800	9200	7900	5300	7000	8900
Day 28	5200	5000	7800	7700	7400	5800	5400	7100	6000
*Lymphocyte, % (Normal range:* 20–40%)									
Day 1	31.5	30.3	43.3	34.2	24.3	28.5	38.2	34.8	44.4
Day 7	31.6	23.7	42.1	34.5	25.2	57.5	40.4	42.9	45.1
Day 14	37.9	27.7	42	44	24.3	39.8	46.3	58.3	24.7
Day 28	38.9	29.8	61.5	38.1	27.3	67.8	40.1	39	53.1
*Erythrocyte sedimentation rate, mm/h* (Normal range: Male < 10; Female < 20 mm/h)									
Day 1	12	8	6	14	12	12	24	6	12
Day 7	14	11	2	15	26	15	17	5	12
Day 14	15	4	2	2	10	10	20	4	15
Day 28	11	2	2	30	16	6	15	4	13
*CRP, mg/L* *(normal range:* < 5 mg/L)									
Day 1	0.8	5.2	0.6	0.7	0.2	12.7	0.4	0.8	1.8
Day 7	1.4	0.2	0.3	1.1	1	2.6	0.4	0.8	0.3
Day 14	0.1	0.3	1.9	1.1	3.5	0.5	0.5	0.6	0.2
Day 28	0.1	0.3	0.5	38.5	0.4	1	2.5	1.1	0.5
*Fibrinogen, mg/dL* *(Normal range:* 150–375 mg/dL)									
Day 1	179	333	222	171	267.5	433.2	282.9	204.6	391.3
Day 7	192	241.6	169.2	181	319.1	319.1	282.9	212.8	288.5
Day 14	253.4	196.9	187.2	165.5	381.9	267.8	277.6	241.6	301.4
Day 28	224.8	232.3	192.6	476.1	249	240.4	253.4	207.5	295.3
*IL-6, ng/L (Normal range:* 0–16.4 ng/L)									
Day 1	35.6	68.1	29	34.3	30.6	125.4	49.3	124.2	36.9
Day 7	10.7	30.6	44.3	26.9	29.4	34.4	64.3	38.1	0.6
Day 14	45.6	15.6	41.8	46.8	19.4	26.2	30.6	8.1	39.3
Day 28	26.9	16.4	23.1	58.1	61.8	20.6	13.2	11.3	18.1
*TNF-α, ng/L (normal range*: 0–29.4 ng/L)									
Day 1	29.6	28.1	19	27.3	27.2	29.6	35.5	33	19.9
Day 7	19.9	23.1	12.6	22.3	24.8	13.4	30.5	27.2	10.9
Day 14	33	13.3	13.8	35.5	15.8	12.8	22.3	19.8	11.4
Day 28	20.7	107.3	19.8	28.1	69.9	16.6	11.8	16.8	19
*IL-1β, ng/L (normal range*: 0–5 ng/L)									
Day 1	0.5	0.5	0.5	0.6	0.5	0.3	0.5	0	0.5
Day 7	0.8	0.3	0.2	1.1	0.5	0	0.1	0.3	0.1
Day 14	0.2	0.2	0	0.3	0.3	0.1	0.1	0.4	0.5
Day 28	0.4	0.1	0.8	0.1	0.1	1	0.1	0.3	0.9
*IFN-γ, ng/L* (normal range: < 8.1 ng/L)									
Day 1	132	148	86	107	104	170	101	179	73
Day 7	86	170	25	123	104	107	86	195	58
Day 14	148	150	17	132	79	51	67	246	29
Day 28	123	30	79	132	19	58	51	33	51

*CRP* C-reactive protein, *IL-6* interleukin 6; *TNF-α* tumor necrosis factor-α, *IL-1β* Interleukin-1β, *IFN-γ *Interferon-γ

At baseline, 3 subjects (TH2, TL3, and C2) showed substantially higher levels of IL-6 that gradually decreased to the normal value on day 28. In 2 subjects (TL1 and TL2), IL-6 increased on day 28. In the 9 subjects, TNF-α was almost normal at baseline and during the study period, except for 2 subjects (TH2 and TL2), in whom TNF-α increased on day 28. No subject with an increasing level of IL-1β was found during the course of the study. However, IFN-γ levels were higher than normal values in all subjects during the course of the investigation. It decreased systematically in 2 subjects (TL2 and C1). A subject (TL1) had IFN-γ values on day 28 higher than baseline, while an irregular trend was observed in the remaining subjects.

## Discussion

This phase 1 trial demonstrated that single TH and TL infusions of DW-MSCs were safe, feasible, and well-tolerated in subjects with low clinical risk COVID-19. No major adverse events related to the administration of DW-MSCs during infusion and at follow-up visits until the end of the study were noted. All clinical parameters were also stable during the study in all groups, and 8 subjects recovered successfully without complications. A subject from the control group had abnormal liver function biomarkers until the end of the study period. The distribution of baseline characteristics, comorbidities, or concomitant treatments between groups was generally balanced. Patient survival was 100%, and there was no discernible difference with respect to clinical outcome between the TH, TL, and C groups.

To our knowledge, this is the first randomized controlled clinical trial to show that DW-MSC infusion is safe and well-tolerated in patients with low clinical risk COVID-19. In our study, only a few TEAEs were related to the clinical progress of COVID-19, such as lobar pneumonia, maculo-popular rash, or increased blood lactate. No signs and symptoms of hypercoagulation such as chest pain, shortness of breath, discomfort in the upper body, ECG abnormalities, thrombotic multi-organ failure, or death were observed in subjects, including those who received placebo. Our findings were consistent with previous reports that the use of MSCs to treat certain diseases in humans has been generally regarded as safe [19, 20]; a review of MSCs therapy studies found an increased risk of fever, but no acute infusion toxicities, infections, thrombotic/embolic events, or malignancy [21]. Our findings were also in line with the previous phase 1 and phase 2a trials of the use of MSCs for ARDS treatment (START) [8, 22] and a phase 1 trial using intravenous human umbilical cord-derived MSCs infusion in patients with moderate and severe COVID-19 that were considered safe and well-tolerated [15].

Several studies have been conducted on exploring the therapeutic efficacy of MSCs in treating patients with COVID-19 (Additional file 1: Table S2). Most of the trials conducted in this regard are not conclusive, though most have reported the safety and efficacy of MSCs. According to PubMed database records, only 10 major studies relevant to clinical trials on MSCs have been reported to date to treat patients with COVID-19 [15–17, 23–29]. Variable results have been reported in these investigations, presumably due to discrepancies in the design of the trial and the quality of the cell product.

In terms of the potential benefits of MSCs, the finding by Leng et al. that the infusion of ACE-2 negative MSCs improved outcomes in the 7 COVID-19 patients is particularly notable [30]. Another notable recent study included 5 COVID-19 patients, 4 of whom were in the intensive care unit, and all required an oxygen mask; most laboratory values and inflammatory markers improved noticeably in a time-dependent manner during the 15-day observation period [26]. Nevertheless, the authors have rightly pointed out the absence of a control group as a major limitation of the work.

However, most of these trials were carried out in patients with severe COVID-19 in the inflammatory phase, and there is no evidence on the effectiveness of MSCs in improving the clinical course of patients with low clinical risk COVID-19 in terms of faster recovery or delay of progression to severity. Furthermore, the source and properties of MSCs can provide significantly diverse results, making it important to research different types of MSCs. With its availability for the industrialization of stem cell technology, the DWP710 stem cell platform has superior competitiveness and high future utilization characteristics. Since 2018, the GLP certification institution has assessed the toxicity and tumorigenicity of DW-MSCs in the human body [31]. Another advantage of DW-MSCs is that it allows for the mass manufacture of verified quality stem cell therapies since it creates stem cells derived from 1 donor cells that have undergone stringent quality checks.

During the clinical course, there were no significant differences between the 3 study groups based on the WHO ordinal scale, NEWS2, PaO_2_/FiO_2_ ratio, or chest X-ray data. Based on the duration of viral shedding, DW-MSCs did not reduce or enhance any parameter of clinical improvements, such as hospital days. Though this clinical study offers good support for the safety of DW-MCSs at both low and high dosages, a previously unknown feature, additional, comprehensive clinical studies are required to draw a statistically robust comparison between the 3 groups.

In terms of inflammatory state, none of the 3 research groups showed any obvious therapeutic benefit. Saleh et al. [26] reported a systematic decrease in various inflammatory markers during the clinical course; however, when compared to the control group, our trial did not reflect any substantial therapeutic advantage of MSCs. We assume that the use of inflammatory markers as a criterion for the better clinical course was not practicable in our investigation since all or most of the subjects presented at the hospital with normal leukocyte counts (4–10 × 10^3^/μL), lymphocyte percentage (20–40%), ESR (20 mm/h), and IL-1 (5 ng/L).

In COVID-19, serum IL-6 is considered to be a physiologically relevant biomarker associated with disease progression, and it has been proposed that IL-6 receptor blocking medicine could aid in the therapeutic improvement in individuals with severe and critical COVID-19 [32]. Furthermore, given the various immune-modulatory pathways of MSCs, a gradual decrease in IL-6 levels has been proposed to be a biologically valid surrogate of treatment success in COVID-19 patients [15]. In our study, despite the lack of a comparable analysis due to the small number of subjects, most of the subjects demonstrated a reduction in IL-6 as their condition improved. Of note, in our study, the 2 subjects with the highest IL-6 level showed the most significant drop in the IL-6 level; however, one of them was from the control group. The cytokine profiles in our study, particularly TNF-α and IL-6, were consistent with the previous report that the levels of inflammation markers varied even between subjects within the same clinical categories and fluctuated dynamically [33].

The most perplexing aspect to interpret was the variation in IFN-γ levels among groups during the clinical course. Most of the subjects had significantly higher levels of IFN-γ even after 28 days. These findings are particularly noteworthy because interferons are frequently touted as a potential treatment modality for COVID-19 [34], because it inhibits virus replication and stimulates cytokine production by T cells [35]. On the other hand, persistently high levels of IFN-γ can worsen systemic inflammation, increasing tissue injury and organ failure [36]. Our results contrast with the recently reported findings of Saleh et al. [26], who observed a systematic decline in the IFN-γ levels. Notably, Lanzoni et al. also reported a significant decrease in IFN-γ levels after MSC therapy; however, in the control group, there was no statistically significant trend [16]. Such factors may partly explain our results; however, given the limitations of the current study as discussed below, we cannot fully elucidate the mechanistic aspects of the observed IFN-γ levels.

Interpretation of the efficacy of MSCs in this study is limited due to the small number of subjects and mild symptoms or low clinical risk. Considering the multiple immune-modulatory mechanisms of MSCs, the efficacy may be more feasible in subjects with moderate to severe COVID-19 and may be most beneficial for individuals with high levels of inflammatory cytokines. The study needs to take into account disease severity; moreover, confounding effects cannot be completely avoided. Because none of our subjects was in critical condition, a direct comparison with previously reported clinical trials, which mainly involved patients with severe COVID-19 infection, is not entirely possible. Clinical trials in severely or critically ill patients are challenging because it is sometimes impossible to determine whether medical occurrences are attributable to the underlying critical illness or the experimental therapy being evaluated. Our study is, in this sense, more controlled than previous studies. More studies are needed in a larger group with subjects of various clinical severity, with more frequent evaluation, and inclusion of more biomarkers that were not measured in our study (e.g., ferritin, d-dimer) will certainly improve the confidence. Although our study has shown that the low and high dose of DW-MSC was safe, it is not clear whether this is also safe in subjects with moderate or severe COVID-19, as MSC products can express variable levels of highly procoagulant tissue factor, compromising the hemocompatibility and safety profile of cells.

## Conclusions

Our clinical trials have provided reliable results regarding the safety of MSCs in low clinical risk COVID-19 subjects treated with MSCs. However, further confirmation of the therapeutic efficacy aspects of MSC will require large-scale randomized controlled trials in subjects with varying severity profiles for COVID-19.

## Supplementary Information

**Additional file 1: Table S1**. Flowchart of Clinical Study. **Table S2**. Published Stem Cell Trials in COVID-19. **Figure S1**. PaO2/FiO2 ratio in the patients with COVID-19 with/without MSCs infusion on days 1, 7, 14, and 28. **Figure S2**. Variation of key inflammatory markers during the clinical course.

## Data Availability

The dataset supporting the conclusions of this article is included within the article (and its additional files).

## Abbreviations

ARDS: Acute respiratory distress syndrome
C: Placebo group
COVID-19: Coronavirus disease of 2019
GMP: Good manufacturing process
ISCT: International Society for Cell & Gene Therapy
IP: Investigational product
NEWS2: National early warning score 2
MeDRA: Medical Dictionary for Regulatory Activities
MSCs: Mesenchymal stem cells
MWCB: Master working cell bank
NCI-CTCAE: National Cancer Institute Common Terminology Criteria for Adverse Events
NIHRD EC: National Institute of Health and Research Development Ethics Committee
NOAEL: No adverse effect level
RT-PCR: Real-time polymerase chain reaction
SARS-CoV-2: Severe acute respiratory syndrome coronavirus 2
TEAE: Treatment-emergent adverse event
TH: Treatment high-dose group
TL: Treatment low-dose group

## References

[1] Moll G, Drzeniek N, Kamhieh-Milz J, Geissler S, Volk H-D, Reinke P (2020). MSC Therapies for COVID-19: importance of patient coagulopathy, thromboprophylaxis, cell product quality and mode of delivery for treatment safety and efficacy. Front Immunol.

[2] Chen Y, Zhang Q, Peng W, Liu D, You Y, Liu X, Tang S, Zhang T (2020). Efficacy and safety of mesenchymal stem cells for the treatment of patients infected with COVID-19: a systematic review and meta-analysis protocol. BMJ Open.

[3] Kyurkchiev D, Bochev I, Ivanova-Todorova E, Mourdjeva M, Oreshkova T, Belemezova K, Kyurkchiev S (2014). Secretion of immunoregulatory cytokines by mesenchymal stem cells. World J Stem Cells.

[4] Sadeghi S, Soudi S, Shafiee A, Hashemi SM (2020). Mesenchymal stem cell therapies for COVID-19: current status and mechanism of action. Life Sci.

[5] Leng Z, Zhu R, Hou W, Feng Y, Yang Y, Han Q, Shan G, Meng F, Du D, Wang S (2020). Transplantation of ACE2-mesenchymal stem cells improves the outcome of patients with COVID-19 pneumonia. Aging Dis.

[6] Chuang H-M, Shih TE, Lu K-Y, Tsai S-F, Harn H-J, Ho L-I (2018). Mesenchymal Stem Cell Therapy of Pulmonary Fibrosis: Improvement with Target Combination. Cell Transplant.

[7] Harrell CR, Sadikot R, Pascual J, Fellabaum C, Jankovic MG, Jovicic N, Djonov V, Arsenijevic N, Volarevic V (2019). Mesenchymal stem cell-based therapy of inflammatory lung diseases: current understanding and future perspectives. Stem Cells Int.

[8] Matthay MA, Calfee CS, Zhuo H, Thompson BT, Wilson JG, Levitt JE, Rogers AJ, Gotts JE, Wiener-Kronish JP, Bajwa EK (2019). Treatment with allogeneic mesenchymal stromal cells for moderate to severe acute respiratory distress syndrome (START study): a randomised phase 2a safety trial. Lancet Respir Med.

[9] Namba F (2019). Mesenchymal stem cells for the prevention of bronchopulmonary dysplasia. Pediatr Int.

[10] Suvakov S, Richards C, Nikolic V, Simic T, McGrath K, Krasnodembskaya A, McClements L (2020). Emerging therapeutic potential of mesenchymal stem/stromal cells in preeclampsia. Curr Hypertens Rep.

[11] Thakkar UG, Trivedi HL, Vanikar AV, Dave SD (2015). Insulin-secreting adipose-derived mesenchymal stromal cells with bone marrow–derived hematopoietic stem cells from autologous and allogenic sources for type 1 diabetes mellitus. Cytotherapy.

[12] Cho J, D'Antuono M, Glicksman M, Wang J, Jonklaas J (2018). A review of clinical trials: mesenchymal stem cell transplant therapy in type 1 and type 2 diabetes mellitus. Am J Stem Cells.

[13] Xu P, Yang X (2019). The efficacy and safety of mesenchymal stem cell transplantation for spinal cord injury patients: a meta-analysis and systematic review. Cell Transplant.

[14] Feng Y, Huang J, Wu J, Xu Y, Chen B, Jiang L, Xiang H, Peng Z, Wang X (2020). Safety and feasibility of umbilical cord mesenchymal stem cells in patients with COVID-19 pneumonia: a pilot study. Cell Prolif.

[15] Meng F, Xu R, Wang S, Xu Z, Zhang C, Li Y, Yang T, Shi L, Fu J, Jiang T (2020). Human umbilical cord-derived mesenchymal stem cell therapy in patients with COVID-19: a phase 1 clinical trial. Signal Transduct Target Ther.

[16] Lanzoni G, Linetsky E, Correa D, Messinger Cayetano S, Alvarez RA, Kouroupis D, Alvarez Gil A, Poggioli R, Ruiz P, Marttos AC (2021). Umbilical cord mesenchymal stem cells for COVID-19 acute respiratory distress syndrome: A double-blind, phase 1/2a, randomized controlled trial. Stem Cells Transl Med.

[17] Shi L, Huang H, Lu X, Yan X, Jiang X, Xu R, Wang S, Zhang C, Yuan X, Xu Z (2021). Effect of human umbilical cord-derived mesenchymal stem cells on lung damage in severe COVID-19 patients: a randomized, double-blind, placebo-controlled phase 2 trial. Signal Transduct Target Ther.

[18] Shahani P, Datta I (2021). Mesenchymal stromal cell therapy for coronavirus disease 2019: which? When? And how much?. Cytotherapy.

[19] Cao Y, Wu H, Zhai W, Wang Y, Li M, Li M, Yang L, Tian Y, Song Y, Li J (2020). A safety consideration of mesenchymal stem cell therapy on COVID-19. Stem Cell Res.

[20] Lohan P, Treacy O, Griffin MD, Ritter T, Ryan AE (2017). Anti-donor immune responses elicited by allogeneic mesenchymal stem cells and their extracellular vesicles: are we still learning?. Front Immunol.

[21] Thompson M, Mei SHJ, Wolfe D, Champagne J, Fergusson D, Stewart DJ, Sullivan KJ, Doxtator E, Lalu M, English SW (2020). Cell therapy with intravascular administration of mesenchymal stromal cells continues to appear safe: An updated systematic review and meta-analysis. EClinicalMedicine.

[22] Wilson JG, Liu KD, Zhuo H, Caballero L, McMillan M, Fang X, Cosgrove K, Vojnik R, Calfee CS, Lee J-W (2015). Mesenchymal stem (stromal) cells for treatment of ARDS: a phase 1 clinical trial. Lancet Respir Med.

[23] Adas G, Cukurova Z, Yasar KK, Yilmaz R, Isiksacan N, Kasapoglu P, Yesilbag Z, Koyuncu ID, Karaoz E (2021). The systematic effect of mesenchymal stem cell therapy in critical COVID-19 patients: a prospective double controlled trial. Cell Transplant.

[24] Hashemian SR, Aliannejad R, Zarrabi M, Soleimani M, Vosough M, Hosseini SE, Hossieni H, Keshel SH, Naderpour Z, Hajizadeh-Saffar E (2021). Mesenchymal stem cells derived from perinatal tissues for treatment of critically ill COVID-19-induced ARDS patients: a case series. Stem Cell Res Ther.

[25] Kouroupis D, Lanzoni G, Linetsky E, Messinger Cayetano S, Wishnek Metalonis S, Leñero C, Stone LD, Ruiz P, Correa D, Ricordi C (2021). Umbilical Cord-derived mesenchymal stem cells modulate TNF and soluble TNF Receptor 2 (sTNFR2) in COVID-19 ARDS patients. Eur Rev Med Pharmacol Sci.

[26] Saleh M, Vaezi AA, Aliannejad R, Sohrabpour AA, Kiaei SZF, Shadnoush M, Siavashi V, Aghaghazvini L, Khoundabi B, Abdoli S (2021). Cell therapy in patients with COVID-19 using Wharton's jelly mesenchymal stem cells: a phase 1 clinical trial. Stem Cell Res Ther.

[27] Sengupta V, Sengupta S, Lazo A, Woods P, Nolan A, Bremer N (2020). Exosomes Derived from Bone Marrow Mesenchymal Stem Cells as Treatment for Severe COVID-19. Stem Cells Dev.

[28] Shu L, Niu C, Li R, Huang T, Wang Y, Huang M, Ji N, Zheng Y, Chen X, Shi L (2020). Treatment of severe COVID-19 with human umbilical cord mesenchymal stem cells. Stem Cell Res Ther.

[29] Xu X, Jiang W, Chen L, Xu Z, Zhang Q, Zhu M, Ye P, Li H, Yu L, Zhou X (2021). Evaluation of the safety and efficacy of using human menstrual blood-derived mesenchymal stromal cells in treating severe and critically ill COVID-19 patients: an exploratory clinical trial. Clin Transl Med.

[30] Leng Z, Zhu R, Hou W, Feng Y, Yang Y, Han Q, Shan G, Meng F, Du D, Wang S (2020). Transplantation of ACE2(-) mesenchymal stem cells improves the outcome of patients with COVID-19 pneumonia. Aging Dis.

[31] Daewoong: bio regenerative industry issue keyword, ‘stem cells’—from COVID-19 treatments to bone regeneration. In: Daewoong Indonesia*.* 2020.

[32] Zhang J, Hao Y, Ou W, Ming F, Liang G, Qian Y, Cai Q, Dong S, Hu S, Wang W (2020). Serum interleukin-6 is an indicator for severity in 901 patients with SARS-CoV-2 infection: a cohort study. J Transl Med.

[33] Han H, Ma Q, Li C, Liu R, Zhao L, Wang W, Zhang P, Liu X, Gao G, Liu F (2020). Profiling serum cytokines in COVID-19 patients reveals IL-6 and IL-10 are disease severity predictors. Emerg Microbes Infect.

[34] Haji Abdolvahab M, Moradi-Kalbolandi S, Zarei M, Bose D, Majidzadeh AK, Farahmand L (2021). Potential role of interferons in treating COVID-19 patients. Int Immunopharmacol.

[35] Levy DE, García-Sastre A (2001). The virus battles: IFN induction of the antiviral state and mechanisms of viral evasion. Cytokine Growth Factor Rev.

[36] Yin K, Gribbin E, Wang H (2005). Interferon-gamma inhibition attenuates lethality after cecal ligation and puncture in rats: implication of high mobility group box-1. Shock (Augusta, Ga).

