# Supplementary material for: Safety of DW-MSC infusion in patients with low clinical risk COVID-19 infection: a randomized, double-blind, placebo-controlled trial
Source: Stem Cell Res Ther. 2022 Apr 1;13:134. doi: 10.1186/s13287-022-02812-4 (PMC8972711; doi:10.1186/s13287-022-02812-4)
Supplement: Supplementary file 1 — Additional file 1: Table S1. Flowchart of Clinical Study. Table S2. Published Stem Cell Trials in COVID-19. Figure S1. PaO2/FiO2 ratio in the patients with COVID-19 with/without MSCs infusion on days 1, 7, 14, and 28. Figure S2. Variation of key inflammatory markers during the clinical course. [file 13287_2022_2812_MOESM1_ESM.docx]

**Table S1. Flowchart of Clinical Study**

| Activity | Screening | Treatment | | End of Treatment | Dropout Visit |
| --- | --- | --- | --- | --- | --- |
| Visit | V1 | V2 | V3 | V4 |  |
| Schedule | Day -7 | Day 1 | Day 2 – Day 28 | Day 28 - end |  |
| Window | Day -7 – Day 1 | - | - | - |  |
| Written consent | √ |  |  |  |  |
| Demographic survey | √ |  |  |  |  |
| Medical history^a^ | √ | √ |  |  |  |
| Antecedent and concomitant drugs^b^ | √ | √ | √ | √ | √ |
| Vital signs^c^ | √ | √ | √ | √ | √ |
| Physical examination | √ | √ | √ | √ | √ |
| Laboratory assessment^d^ | √ | √ | √ | √ | √ |
| Pregnancy test^e^ | √ |  |  | √ | √ |
| Electrocardiography (ECG)^f^ | √ |  |  | √ | √ |
| Chest X-ray test^g^ | √ |  | √ | √ | √ |
| PaO_2_, FiO_2_ test^h^ | √ | √ | √ | √ | √ |
| Ordinal scale for clinical improvement^i^ | √ | √ | √ | √ | √ |
| National Early Warning Score^j^ | √ | √ | √ | √ | √ |
| Confirmation of inclusion/ exclusion criteria | √ | √ |  |  |  |
| Randomization |  | √ |  |  |  |
| Prescription of investigation product |  | √ |  |  |  |
| Confirmation for adverse events | √ | √ | √ | √ | √ |

^a^Investigate the medical and surgical history that occurred only within 6 months of screening criteria, or during screening and baseline.

^b^Antecedent drugs are collected for the administered drugs within 4 weeks based on the date of written consent on Visit 1.

^c^Vital signs: after resting for 5 minutes, measure blood pressure (systolic/diastolic), pulse, and body temperature in a sitting position.

^d^The laboratory test items are as follows: 1. hematology (RBC, hemoglobin, hematocrit, platelet, WBC, neutrophil, lymphocyte, monocyte, eosinophil, basophil); 2. Serum chemistry (Na, K, Cl, creatinine, uric acid, BUN, ALT, AST, ALP, LDH, γ-GTP, total bilirubin, albumin, total protein, total cholesterol, TG, fasting glucose, eGFR(calculation), ESR, CRP); 3. serum coagulation (PT(INR), aPTT, fibrinogen); 4. urinalysis (pH, specific gravity, albumin, bilirubin, glucose, urobilinogen, ketone, RBC, WBC); and 5. cytokines (IL-6, TNF-α, IL-1β, IF-γ). All laboratory test results, including screening tests, use only the test results performed on the visit. During the screening, the results of the inflammation markers test should be secured before the (random) assignment, and then, the progress of the COVID-19 is evaluated by checking the inflammation markers on the visits on Day 7, 14, and 28

^e^Pregnancy test by serum test (pregnancy test is exempt for women who are medically unable to become pregnant). However, if Visit 1 and Visit 2 are performed on the same date, the pregnancy test (serum test) should be additionally conducted before administration of the IPeven if there are test results within 2 weeks from the date. After that, the study is conducted at the last visit (Visit 4).

^f^During the screening test, the results of the electrocardiography test should be secured before the (random) assignment to replace the results within 1 week before the (random) assignment. After that, the study is conducted at Visit 2 and at the last visit (Visit 4).

^g^During the screening, chest X-ray test results should be secured before the (random) assignment, and then, the progress of COVID-19 through chest X-ray test is evaluated on the visits on Day 7, 14, and 28.

^h^During the screening, PaO_2_ and FiO_2_ test results should be secured before (random) assignment, and then, the progress of COVID-19 through PaO_2_ and FiO_2_ tests is evaluated on the visits on Day 1, 3, 7, 10, 14, and 28

^i^During the screening, the results of the ordinal scale test should be secured before the (random) assignment, and the progress of COVID-19 is evaluated by the ordinal scale test for each visit. Ordinal Scale for Clinical Improvement : 0 (Uninfected)), 1 (Ambulatory), 2: (Limitation of activities); 3 (Hospitalized, no oxygen therapy), 4 (Mild Disease, Oxygen by mask or nasal prongs), 5 (Non-invasive ventilation or high-flow oxygen), 6 (Intubation and mechanical ventilation) , 7 (Ventilation + additional organ support-ECMO, CRRT, pressors) , 8 (Death) [1]

^j^During screening, the National Early Warning Score 2 (NEWS2) test result should be secured before the (random) assignment, and the progress of COVID-19 is evaluated through the NEWS2 test on the visits on Day 7, 14, and 28.

NEWS2 [2, 3]:

| **Score** | **3** | **2** | **1** | **0** | **1** | **2** | **3** |
| --- | --- | --- | --- | --- | --- | --- | --- |
| %SpO_2_ | ≤ 91 | 92-93 | 94-95 | ≥ 96 |  |  |  |
| Supplemental oxygen |  | Yes |  | No |  |  |  |
| Heart rate | ≤ 40 |  | 41-50 | 51-90 | 91-110 | 111-130 | ≥ 131 |
| Systolic blood pressure | ≤ 90 | 91-100 | 101-110 | 111-219 |  |  | ≥ 220 |
| Respiratory rate | ≤ 8 |  | 9-11 | 12-20 |  | 21-24 | ≥ 25 |
| Body temperature | ≤ 35.0 |  | 35.1-36.0 | 36.1-38.0 | 38.1-39.0 | ≥39.1 |  |
| Central nervous system level |  |  |  | A |  |  | V, P, U |
|  | A: Alert, V: Reaction to Voice, P: Reaction to Pain, U: Unresponsive | | | | | | |

Interpretation of NEWS2:

| **Points** | **Risk** | **Interpretation** |
| --- | --- | --- |
| 0-4 (but no 3 point risk item) | Low clinical risk | Ward-based response |
| 3-4 (including one 3 point risk item) | Low-medium clinical risk | Urgent ward-based response |
| 5-6 | Medium clinical risk | Key threshold for urgent response |
| 7~20 | High clinical risk | Urgent or emergency response |

**References**:

1. Chadjichristos CE, Scheckenbach KE, van Veen TA, Richani Sarieddine MZ, de Wit C, Yang Z, Roth I, Bacchetta M, Viswambharan H, Foglia B *et al*: Endothelial-specific deletion of connexin40 promotes atherosclerosis by increasing CD73-dependent leukocyte adhesion. *Circulation* 2010, 121(1):123-131.

2. Spagnolli W, Rigoni M, Torri E, et al. Application of the National Early Warning Score (NEWS) as a stratification tool on admission in an Italian acute medical ward: A perspective study. *Int J Clin Pract* 2017;71(3-4).

3. Royal College of Physicians. National Early Warning Score (NEWS) 2: Standardizing the assessment of acute-illness severity in the NHS. Updated report of a working party. London: RCP 2017. Available at:

https://www.rcplondon.ac.uk/projects/outputs/national-early-warning-score-NEWS-2 (Accessed on February 21, 2020).

**Table S2. Published Stem Cell Trials in COVID-19**

|  | **Authors** | **Journal** | **Title** |
| --- | --- | --- | --- |
| 1 | Liu et al. | medRxiv (2020.02.27) | Clinical features and progression of acute respiratory distress syndrome in coronavirus disease 2019 |
| 2 | Qi et al. | medRxiv (2020.03.03) | Epidemiological and clinical features of 2019-nCoV acute respiratory disease cases in Chongqing municipality, China: a retrospective, descriptive, multiple-center study |
| 3 | Arentz et al. | JAMA (2020.03.19) | Characteristics and Outcomes of 21 Critically Ill Patients With COVID-19 in Washington State |
| 4 | Deng et al. | Chinese Medical Journal (2020.03.20) | Clinical characteristics of fatal and recovered cases of coronavirus disease 2019 (COVID-19) in Wuhan, China |
| 5 | Leng et al. | Aging and Disease (2020) | Transplantation of ACE2- Mesenchymal Stem Cells Improves the Outcome of Patients with COVID-19 Pneumonia |
| 6 | Zhang et al. | medRxiv (2020.03.26) | COVID-19 infection induces readily detectable morphological and inflammation-related phenotypic changes in peripheral blood monocytes, the severity of which correlate with patient outcome |
| 7 | Wu et al. | Cell (2018) | Intrinsic Immunity Shapes Viral Resistance of Stem Cells |
| 8 | Chan et al. | PNAS (2016) | Human mesenchymal stromal cells reduce influenza A H5N1-associated acute lung injury in vitro and in vivo |
| 9 | Zhou et al. | Lancet (2020.03.11) | Clinical course and risk factors for mortality of adult inpatients with COVID-19 in Wuhan, China: a retrospective cohort study |
| 10 | Wu et al. | JAMA Internal Medicine (2020.03.13) | Risk Factors Associated With Acute Respiratory Distress Syndrome and Death in Patients With Coronavirus Disease 2019 Pneumonia in Wuhan, China |
| 11 | Hirsch et al. | Pain Physician. (2020.03) | Expanded Umbilical Cord Mesenchymal Stem Cells (UC-MSCs) as a Therapeutic Strategy in Managing Critically Ill COVID-19 Patients: The Case for Compassionate Use |
| 12 | Giacomo Lanzoni et al. | Stem Cells Translation Medicine  (2021.01) | Umbilical cord mesenchymal stem cells for COVID‐19 acute respiratory distress syndrome: A double‐blind, phase 1/2a, randomized controlled trial |
| 13 | Arefeh Basiri et al. | Stem Cell Reviews and Reports (2021.02) | Stem Cell Therapy Potency in Personalizing Severe COVID-19 Treatment |
| 14 | Claudia Musial and Magdalena Gorska-Ponikowska | Stem Cell Research  (2021.04) | Medical progress: Stem cells as a new therapeutic strategy for COVID-19 |
| 15 | Lei Shi et al. | Signal transduction and targeted therapy  (2021.02) | Effect of human umbilical cord-derived mesenchymal stem cells on lung damage in severe COVID-19 patients: a randomized, double-blind, placebo-controlled phase 2 trial |
| 16 | Na Song et al. | Stem Cells  (2021. 02) | Mesenchymal stromal cell immunomodulation: In pursuit of controlling COVID‐19 related cytokine storm |
| 17 | Christopher J. Rogers et al. | Journal of Translational Medicine (2020.05) | Rationale for the clinical use of adipose-derived mesenchymal stem cells for COVID-19 patients |
| 18 | Helene Häberle et al. | Journal of Intensive Care Medicine  (2021.03) | Mesenchymal Stem Cell Therapy for Severe COVID-19 ARDS |
| 19 | Angliana Chouw et al. | Regen Eng Transl Med. (2021.03) | Potency of Mesenchymal Stem Cell and Its Secretome in Treating COVID-19 |
| 20 | Qiqi Cai et al. | Stem Cells Dev. (2021.03) | Research Progress of Mesenchymal Stem Cell Therapy for Severe COVID-19 |
| 21 | Fengtao Wei et al. | Clinics (Sao Paulo)  (2021.05) | Efficacy and safety of umbilical cord mesenchymal stem cells for the treatment of patients with COVID-19 |
| 22 | Rachele Ciccocioppo et al. | Stem Cell Research & Therapy  (2021. 06) | The immune modulatory effects of umbilical cord-derived mesenchymal stromal cells in severe COVID-19 pneumonia |
| 23 | Ismail Hadisoebroto Dilogo et al. | Stem Cells Translation Medicine  (2021. 06) | Umbilical cord mesenchymal stromal cells as critical COVID-19 adjuvant therapy: A randomized controlled trial |
| 24 | Miguel Reyes et al. | Science Translational Medicine (2021. 06) | Plasma from patients with bacterial sepsis or severe COVID-19 induces suppressive myeloid cell production from hematopoietic progenitors in vitro |
| 25 | Dhavan Sharma & Feng Zhao | npj Regenerative Medicine (2021.06) | Updates on clinical trials evaluating the regenerative potential of allogenic mesenchymal stem cells in COVID-19 |
| 26 | D. Kouroupis et al. | Eur Rev Med Pharmacol Sci  (2021.06) | Umbilical Cord-derived Mesenchymal Stem Cells modulate TNF and soluble TNF Receptor 2 (sTNFR2) in COVID-19 ARDS patients |
| 27 | G Adas et al. | Cell Transplant  . (2021.06) | The Systematic Effect of Mesenchymal Stem Cell Therapy in Critical COVID-19 Patients: A Prospective Double Controlled Trial |
| 28 | Vuong Cat Khanh et al. | Stem Cells and Development (2021.07) | Wharton's Jelly Mesenchymal Stem Cell-Derived Extracellular Vesicles Reduce SARS-CoV2-Induced Inflammatory Cytokines Under High Glucose and Uremic Toxin Conditions |
| 29 | Alice Valentin-Torres et al. | Scientific Reports (2021.06) | Multipotent adult progenitor cells induce regulatory T cells and promote their suppressive phenotype via TGFβ and monocyte-dependent mechanisms |
| 30 | Mahshid Saleh et al. | Stem cell research & therapy  (2021.07) | Cell therapy in patients with COVID-19 using Wharton's jelly mesenchymal stem cells: a phase 1 clinical trial. |
| 31 | O Ercelen N et al. | Stem Cell Rev Rep  (2021.07) | Clinical experience on umbilical cord mesenchymal stem cell treatment in 210 severe and critical COVID-19 cases in Turkey |
| 32 | Chen, K.G. et al. | Nature Cell Biology (2021.08) | Studying SARS-CoV-2 infectivity and therapeutic responses with complex organoids |
| 33 | Mark M Zaki et al. | Science Advances (2021.08) | Cell therapy strategies for COVID-19: Current approaches and potential applications |
| 34 | Abdelgawad M et al. | Stem Cell Res Ther.  (2021. 08) | Mesenchymal stem cell-based therapy and exosomes in COVID-19: current trends and prospects |
| 35 | FELICE D’AGNILLO et al. | SCIENCE TRANSLATIONAL MEDICINE  (2021. 10) | Lung epithelial and endothelial damage, loss of tissue repair, inhibition of fibrinolysis, and cellular senescence in fatal COVID-19 |
| 36 | Mebarki, Miryam et al. | Stem cell research & therapy  (2021. 11) | Development of a human umbilical cord-derived mesenchymal stromal cell-based advanced therapy medicinal product to treat immune and/or inflammatory diseases. |
| 37 | Cloer, Caryn et al. | PLoS One  (2021. 11) | Mesenchymal stromal cell-derived extracellular vesicles reduce lung inflammation and damage in nonclinical acute lung injury: Implications for COVID-19 |

**Figure S1. PaO_2_/FiO_2_ ratio in the patients with COVID-19 with/without MSCs infusion on days 1, 7, 14 and 28**

**Figure S2. Variation of key inflammatory markers during the clinical course**
